# Supplementary material for: Low yield and abiotic origin of N2O formed by the complete nitrifier Nitrospira inopinata
Source: Nat Commun. 2019 Apr 23;10:1836. doi: 10.1038/s41467-019-09790-x (PMC6478695; doi:10.1038/s41467-019-09790-x)
Supplement: Supplementary file 1 — Supplementary Information [file 41467_2019_9790_MOESM1_ESM.pdf]

Low yield and abiotic origin of N_2O formed by the complete nitrifier *Nitrospira inopinata*

Kits et al.

Supplementary Information

Supplementary Figure 1: **Effect of NH_4^+ concentration on NO production by *N. inopinata*.**

Supplementary Figure 2: **Instantaneous O_2 consumption and NO production during NH_3 oxidation by *N. europaea* ATCC 19718.**

Supplementary Figure 3: **Protein abundance levels of *Nitrospira inopinata* during growth on ammonia.**

Supplementary Figure 4: **Instantaneous O_2 consumption and NO production during NO_2^- oxidation by *N. moscoviensis*.**

Supplementary Figure 5: **Effect of the NO scavenger PTIO on *N. inopinata* and *N. moscoviensis*.**

Supplementary Figure 6: **Instantaneous O_2 consumption and NO production during NH_3 oxidation by *N. inopinata*.**

Supplementary Figure 7: **Influence of O_2 limitation on N_2O production by *N. moscoviensis*.**

Supplementary Figure 8: **Instantaneous O_2 consumption and NO production during NO_2^- oxidation by *N. inopinata*.**

Supplementary Figure 9: **Instantaneous O_2 consumption and N_2O production during NH_3 oxidation by *N. inopinata*.**

Supplementary Note 1: **Gene inventory for NO_x metabolism of nitrifiers**

Supplementary References

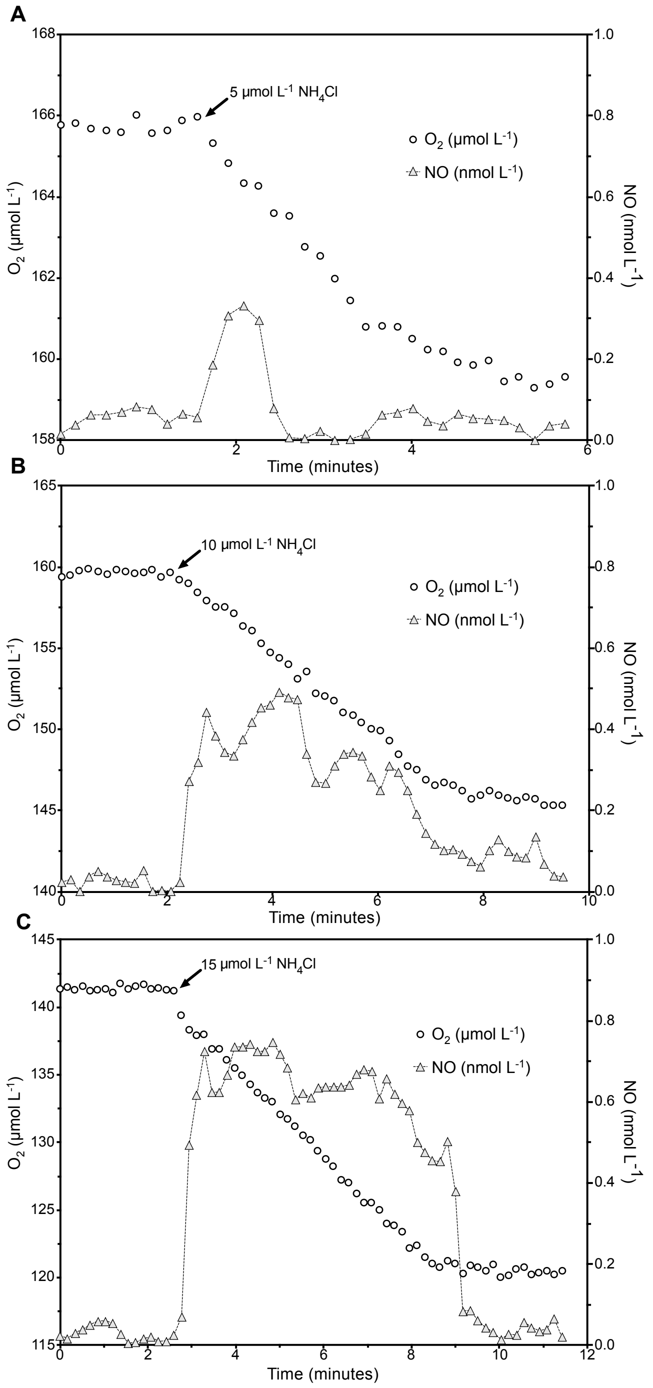

Supplementary Figure 1. Effect of NH_4^+ concentration on NO production by *N. inopinata*.
Dissolved O_2 is shown in open circles and dissolved NO in filled grey triangles. Experiments were performed in a 10 mL MR chamber fitted with an O_2 and NO microsensor. The arrow marks the addition of 5 µM (Panel A), 10 µM (Panel B), or 15 µM (Panel C) mM NH_4Cl into the

MR chamber; the same sample was measured repeatedly. No NO formation from NH_4+ was measurable in oxic sterile media controls containing heat-killed biomass. Source data are provided as a Source Data file.

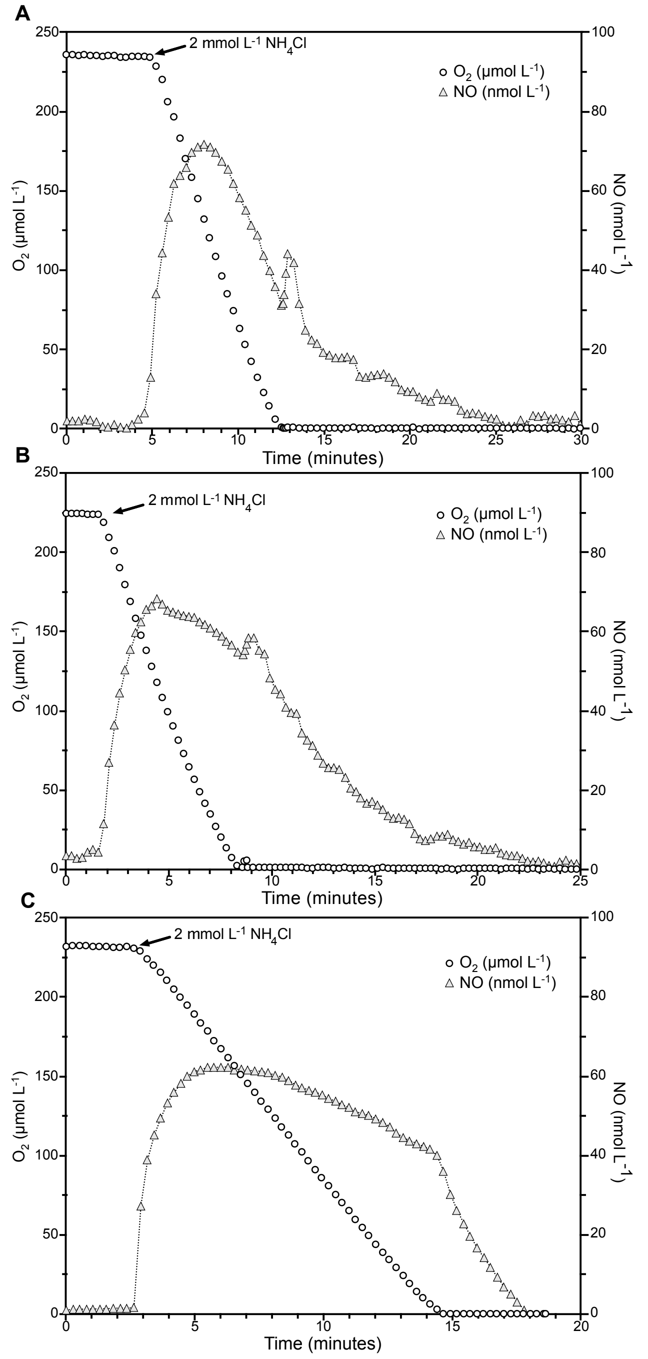

Supplementary Figure 2. Instantaneous O_2 consumption and NO production during NH_3 oxidation by *N. europaea* ATCC 19718. Dissolved O_2 is shown in open circles and dissolved NO in filled grey triangles. Experiments were performed in a 10 mL microrespiration (MR)

chamber fitted with an O_2 and NO microsensor. The arrow marks the addition of 2 mM NH_4Cl into the MR chamber. A total of 3×10^9 cells were used for each of the biological replicates (n = 3), which corresponds to ~3 times less biomass per experiment than used for comparable experiments with *N. inopinata* in Fig. 3 and Supplementary Figure 6. Panels A, B, and C correspond to the three biological replicates (n = 3). No NO formation from NH_4^+ was measurable in oxic sterile media controls containing heat-killed biomass. Source data are provided as a Source Data file.

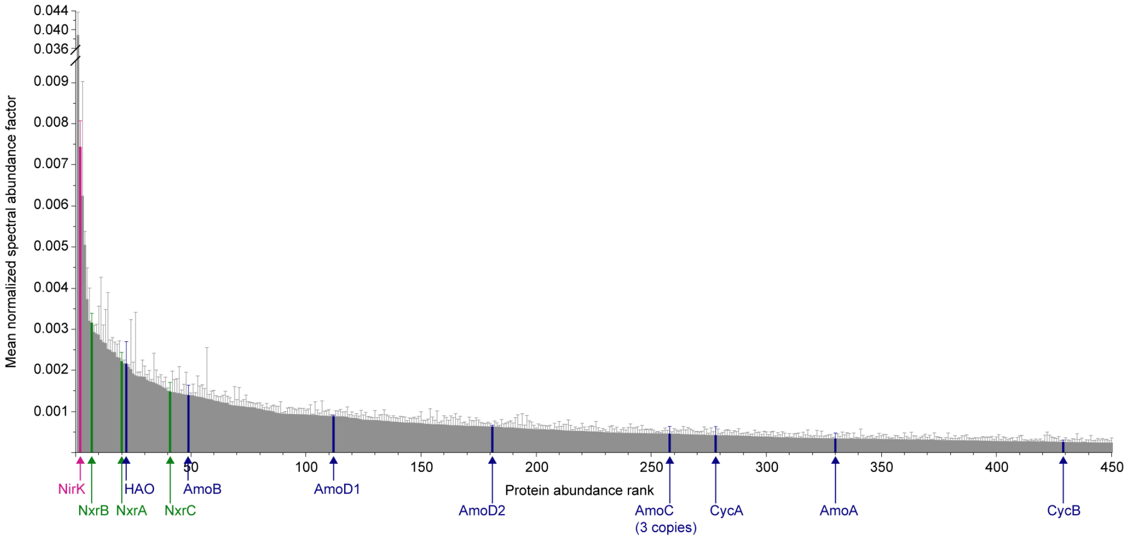

Supplementary Figure 3. Protein abundance levels of *Nitrospira inopinata* during growth on ammonia. Displayed are the 450 most abundant proteins from *N. inopinata*, after incubation with 1 mM ammonium for 48 h under aerobic conditions, in the metaproteome from a completely nitrifying enrichment culture that contained *N. inopinata* as the only ammonia and nitrite oxidizer[13]. Blue arrows and labels indicate key proteins for ammonia oxidation, green arrows and labels indicate key proteins for nitrite oxidation, and magenta highlights copper-containing nitrite reductase (NirK). Columns show the mean normalized spectral abundance factor (NSAF), error bars show 1 s.d. of n=4 biological replicates. In total 1,083 proteins in the metaproteome were unambiguously assigned to *N. inopinata*. Only one of four putative NXR gamma subunits (NxrC) was among the top 450 expressed proteins. The other three NxrC candidates ranked at positions 561, 605, and 931. The AmoE1 protein was ranked at position 520, and HaoB at position 653. Abbreviations: Amo, ammonia monooxygenase (with subunits A to D); CycA, cytochrome c_{554}; CycB, cytochrome c_{m552}; HAO, hydroxylamine dehydrogenase; Nir, nitrite reductase; Nxr, nitrite oxidoreductase (with subunits A to C). Figure modified from reference[13].

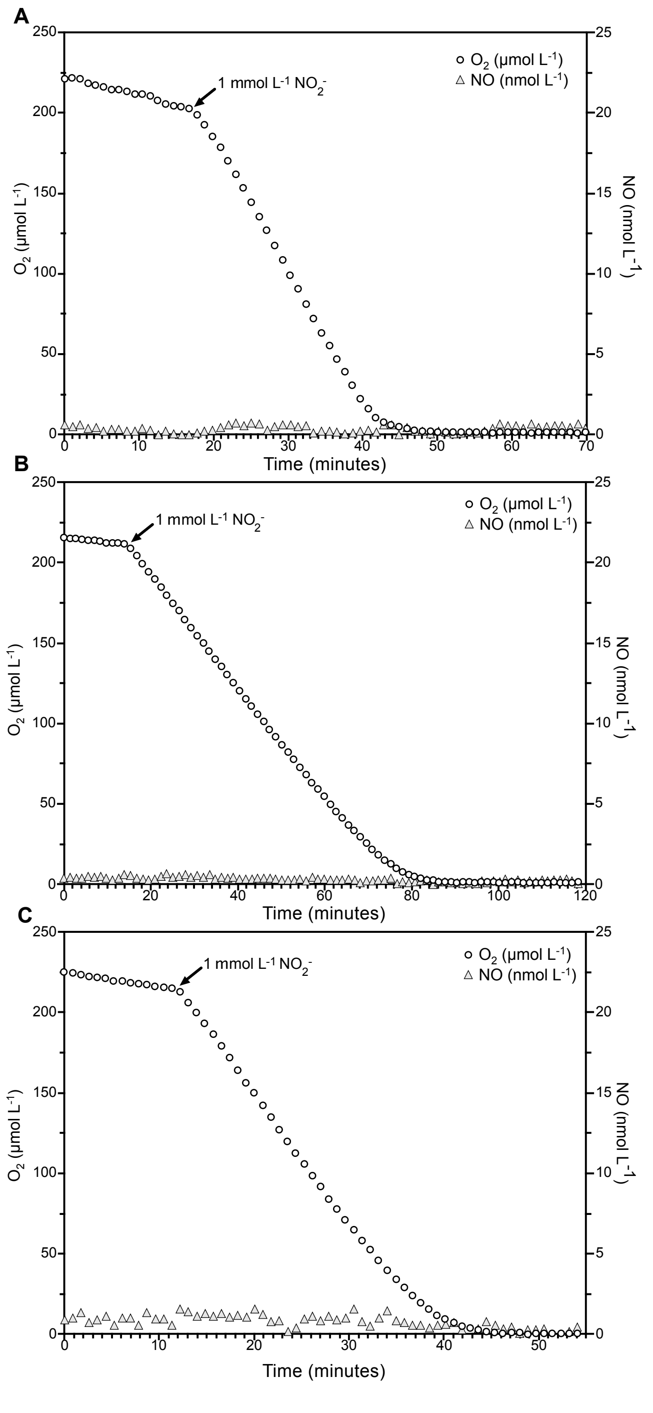

Supplementary Figure 4. Instantaneous O_2 consumption and NO production during NO_2^- oxidation by *N. moscoviensis*. Dissolved O_2 is shown in open circles and dissolved NO in filled grey triangles. Panels A, B, and C correspond to individual experiments from three (n = 3) biological replicates. Experiments were performed in a 10 mL microrespiration (MR) chamber

fitted with an O_2 and NO microsensor. The arrow marks the addition of 1 mM NO_2^- into the MR chamber. No NO formation from NO_2^- was measurable in oxic sterile media controls containing heat-killed biomass. Source data are provided as a Source Data file.

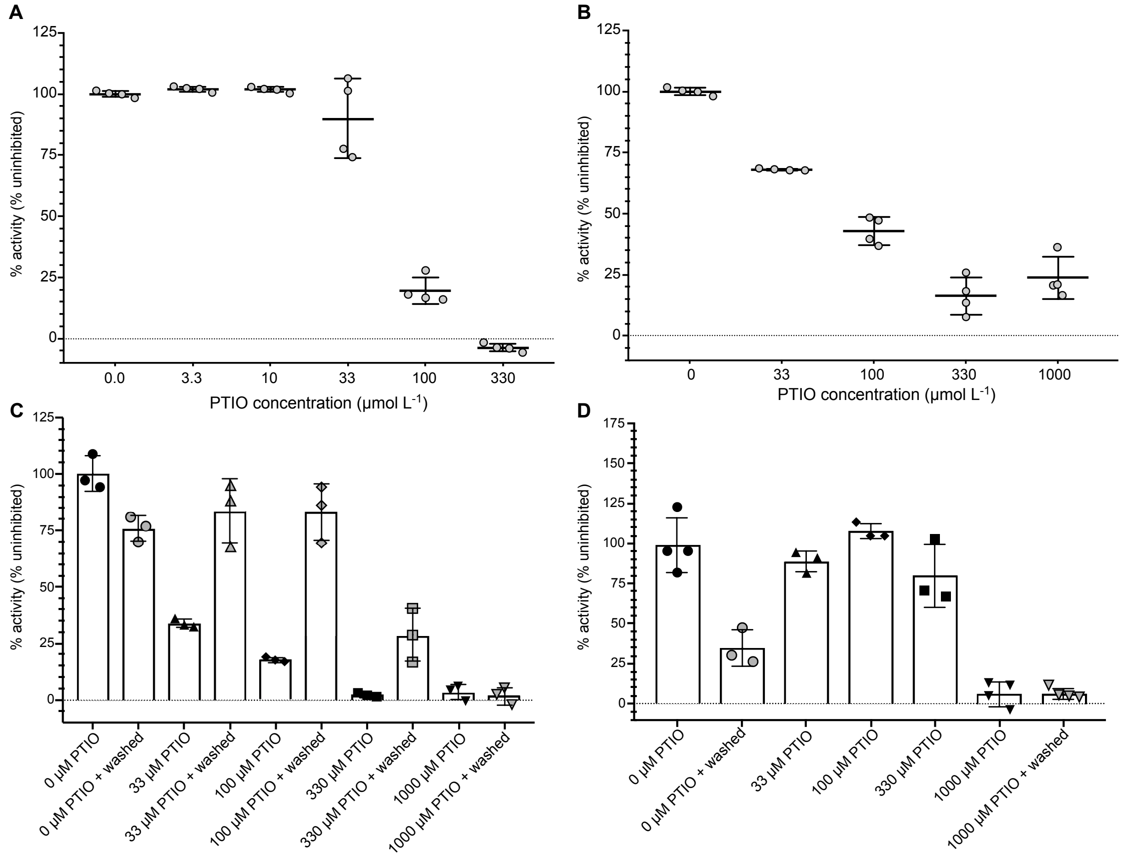

Supplementary Figure 5. Effect of the NO scavenger PTIO on *N. inopinata* and *N. moscoviensis*. Panels A and B indicate the influence of PTIO on batch culture substrate oxidation activity throughout growth in *N. inopinata* (Panel A) and *N. moscoviensis* (Panel B). Panels C and D show the influence of PTIO on substrate-dependent instantaneous O_2 consumption (determined using micro-respirometry) in *N. inopinata* (Panel C) and *N. moscoviensis* (Panel D). For Panels C and D only, cultures which were highly (>50%) inhibited by PTIO were re-harvested and washed as described in the Material and Methods and % activity was remeasured; the activity (%) of all washed cells was normalized to the unwashed 0 µM PTIO control. For all panels, activity (%) shows the difference in rate of linear substrate oxidation (or O_2 consumption) between the non-inhibited control cultures and cultures that were exposed to various concentrations of PTIO; 100% activity signifies no measurable inhibition

while 0% activity indicates total inhibition of activity. No activity was measurable in abiotic and dead cell controls. Center lines indicate the mean and error bars show the standard deviation of the mean for 4 (Panels A and B) or 3 (Panels C and D) biological replicates. The electron donor was always 1 mM NH_3 for *N. inopinata* and 1 mM NO_2^- for *N. moscoviensis*. Source data are provided as a Source Data file.

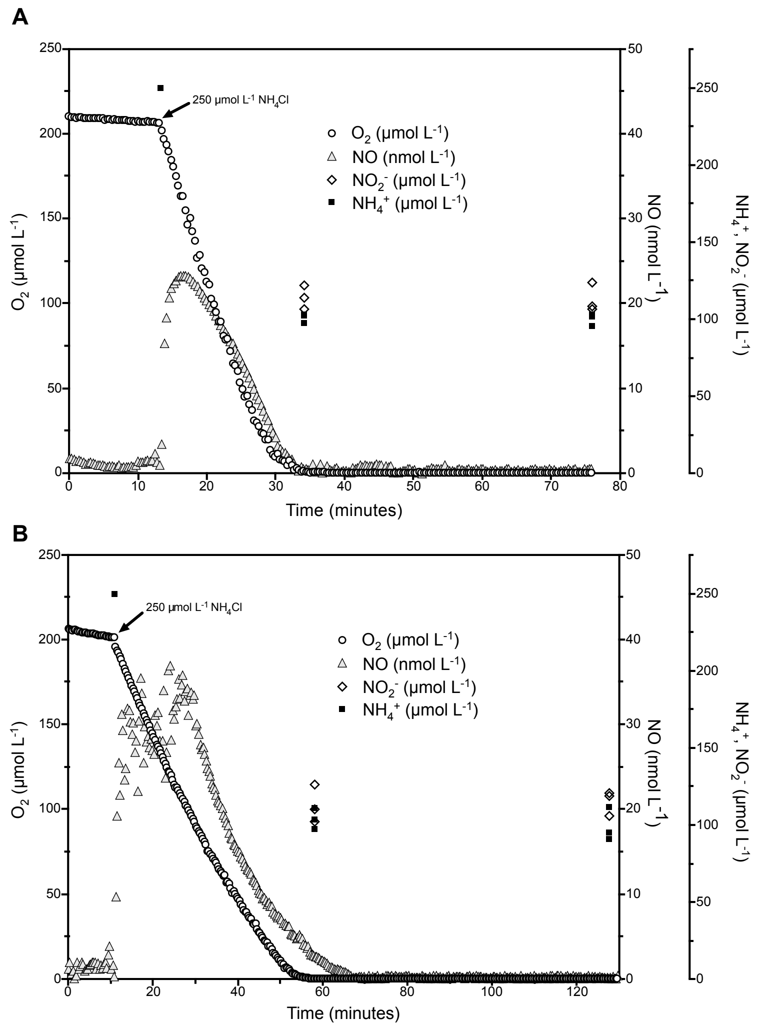

Supplementary Figure 6. Instantaneous O_2 consumption and NO production during NH_3 oxidation by *N. inopinata*. Dissolved O_2 is shown in open circles, dissolved NO in filled grey triangles, NO_2^- in open diamonds, and NH_4^+ in filled squares. The NH_4^+ concentration immediately after injection for each replicate was inferred from the injected volume of a stock NH_4Cl solution, otherwise NO_2^- and NH_4^+ concentrations were determined in 3 technical replicates (n = 3) for each biological replicate. Panels A and B correspond to two biological replicates; a third biological replicate is shown in Fig. 3. Experiments were performed in a microrespiration (MR) chamber fitted with O_2 and NO microsensors. The arrow marks the addition of 250 µM NH_4Cl into the MR chamber. About 110 µM residual NO_2^- was present in

the chamber once O_2 levels dropped below the limit of detection (300 nM). No NO formation from NH_4^+ was measurable in sterile media controls containing the same amount of heat-killed biomass of *N. inopinata*. Source data are provided as a Source Data file.

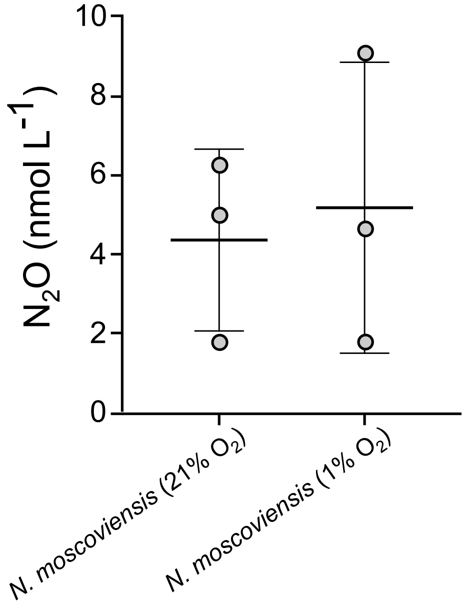

Supplementary Figure 7. Influence of O_2 limitation on N_2O production by *N. moscoviensis*. Accumulation of N_2O in the headspace is shown in nmol L^{-1} above atmospheric N_2O. The black bar indicates N_2O accumulation in cultures of *N. moscoviensis* incubated at atmospheric O_2 concentrations whereas the grey bar indicates N_2O accumulation in cultures incubated at hypoxic (~1.0% at the start of the experiment) O_2 concentrations. The amount of biomass at t = 0 was the same in the oxic and hypoxic vials. The same amount of substrate was consumed in both treatments (1 mM NO_2^-). Center lines indicate the mean and error bars show the standard deviation of the mean for three (n = 3) biological replicates. Source data are provided as a Source Data file.

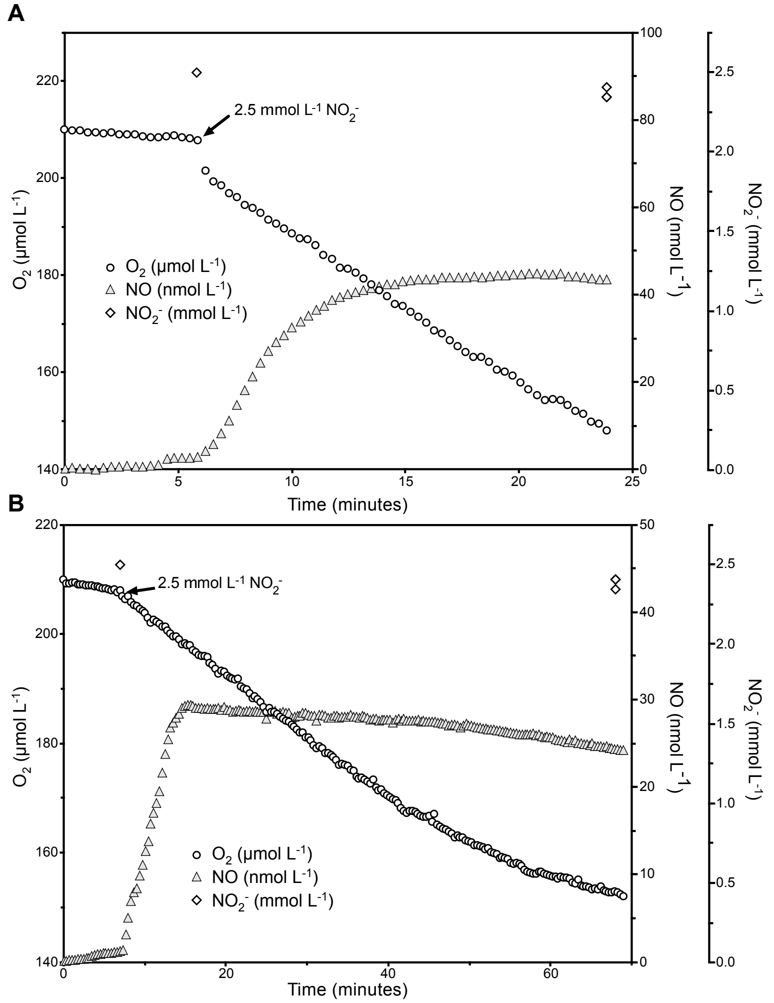

Supplementary Figure 8. Instantaneous O_2 consumption and NO production during NO_2^- oxidation by *N. inopinata*. Dissolved O_2 is shown in open circles, dissolved NO in filled grey triangles, and NO_2^- in open diamonds. The NO_2^- concentration immediately after injection for each replicate was inferred from the injected volume of a stock $NaNO_2$ solution, otherwise NO_2^- concentrations were determined in 3 technical replicates (n = 3) for each biological replicate. Panels A and B correspond to two biological replicates; a third biological replicate is shown in Fig. 4. Experiments were performed in a 10 mL microrespiration (MR) chamber fitted with an O_2 and NO microsensors. The arrow marks the addition of 2.5 mM NO_2^- into the MR chamber.

No NO formation from NO_2^- was measurable in oxic sterile media controls containing heat-killed biomass. Source data are provided as a Source Data file.

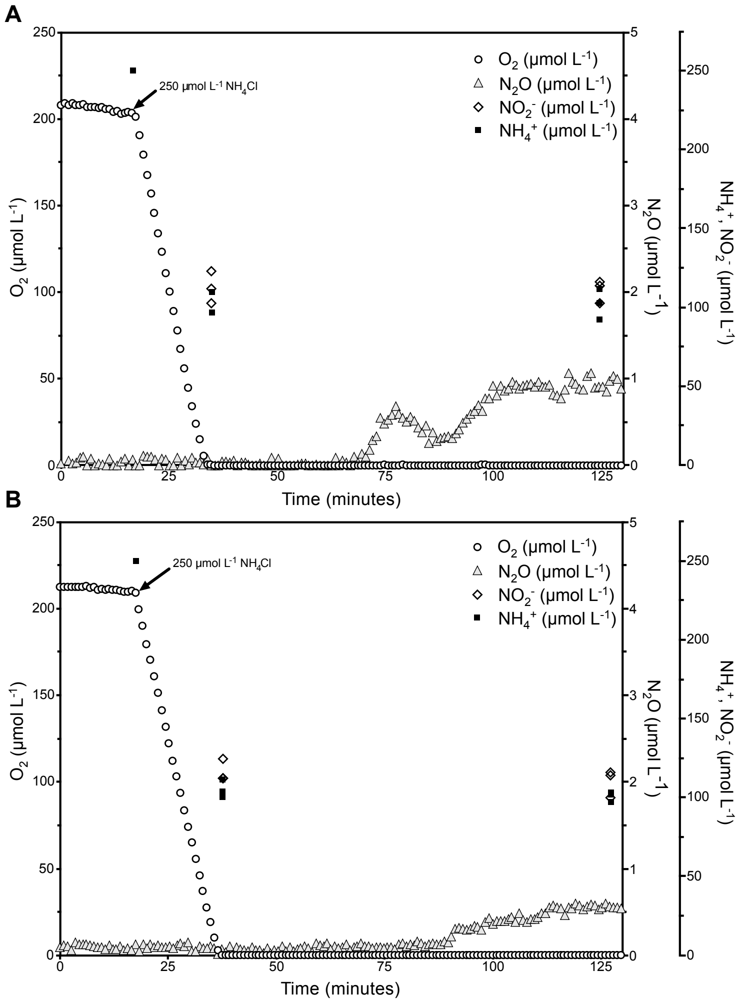

Supplementary Figure 9. Instantaneous O_2 consumption and N_2O production during NH_3 oxidation by *N. inopinata*. Panels A and B correspond to two biological replicates; a third biological replicate is shown in Fig. 5. Dissolved O_2 is shown in open circles, dissolved N_2O in filled grey triangles, NO_2^- in open diamonds, and NH_4^+ in filled squares. The NH_4^+ concentration immediately after injection for each replicate was inferred from the injected volume of a stock NH_4Cl solution, otherwise NO_2^- and NH_4^+ concentrations were determined in 3 technical replicates (n = 3) for each biological replicate. Experiments were performed in a 10 mL microrespiration (MR) chamber fitted with O_2 and N_2O microsensors. The arrow marks the

addition of 250 µM NH_4Cl into the MR chamber. About 110 µM residual NO_2^- was present in the chamber once O_2 reached below the detectable levels. Source data are provided as a Source Data file.

Supplementary Note 1:

Gene inventory for NO_x metabolism of nitrifiers

Dissimilatory nitrite reductase NirK. Of the two known non-homologous dissimilatory nitrite reductases (NirS and NirK), only the copper-containing dissimilatory nitrite reductase *nirK* is encoded in the genomes of nitrifiers. Our extended comparative genome analysis further supports previous observations that *nirK* genes are not found in all AOB genomes[1–3]. In this context it is interesting to note that in AOB *nirK* is not required for NO_2^- reduction to NO and this even holds true for AOB that do possess the gene[4–6], strongly suggesting the existence of novel dissimilatory nitrite reductases in these organisms. In contrast to AOB, AOA lack a canonical HAO and the most current physiological models postulate that NO is an active and necessary co-substrate during the oxidation of NH_2OH to NO_2^- or that NO is the product of NH_2OH oxidation by either NirK or an anchored PEFG-CTERM domain-containing Cu metalloenzyme[7,8]. In these models, NO is either produced or oxidized by NirK that is encoded in the genome of most AOA and was found highly expressed in their transcriptomes and proteomes[8–10]. However, recent work on thermophilic, autotrophic, archaeal ammonia oxidizers shows that these microbes do not possess *nirK* and it has been speculated that they obtain NO from metabolic partner organisms[11,12].

Supplementary References:

1. Kozlowski, J. A., Dimitri Kits, K. & Stein, L. Y. Comparison of nitrogen oxide metabolism

among diverse ammonia-oxidizing bacteria. *Front. Microbiol.* **7**, (2016).

2. Kozlowski, J. A., Kits, K. D. & Stein, L. Y. Genome sequence of *Nitrosomonas communis* strain Nm2, a mesophilic ammonia-oxidizing bacterium isolated from mediterranean soil. *Genome Announc.* **4**, (2016).

3. Hayatsu, M. et al. An acid-tolerant ammonia-oxidizing γ-proteobacterium from soil. *ISME J.* **11**, 1130–1141 (2017).

4. Kozlowski, J. A., Price, J. & Stein, L. Y. Revision of N_2O-producing pathways in the ammonia-oxidizing bacterium *Nitrosomonas europaea* ATCC 19718. *Appl. Environ. Microbiol.* **80**, 4930–4935 (2014).

5. Kozlowski, J. A., Dimitri Kits, K. & Stein, L. Y. Comparison of nitrogen oxide metabolism among diverse ammonia-oxidizing bacteria. *Front. Microbiol.* **7**, (2016).

6. Hayatsu, M. et al. An acid-tolerant ammonia-oxidizing γ-proteobacterium from soil. *ISME J.* **11**, 1130–1141 (2017).

7. Kozlowski, J. A., Stieglmeier, M., Schleper, C., Klotz, M. G. & Stein, L. Y. Pathways and key intermediates required for obligate aerobic ammonia-dependent chemolithotrophy in bacteria and Thaumarchaeota. *ISME J.* **10**, 1836–1845 (2016).

8. Carini, P., Dupont, C. L. & Santoro, A. E. Patterns of thaumarchaeal gene expression in culture and diverse marine environments. *Environ. Microbiol.* (2018). https://doi.org/10.1111/1462-2920.14107.

9. Kerou, M. et al. Proteomics and comparative genomics of *Nitrososphaera viennensis* reveal the core genome and adaptations of archaeal ammonia oxidizers. *Proc. Natl. Acad. Sci. USA* **113**, E7937–E7946 (2016).

10. Qin, W. et al. Stress response of a marine ammonia-oxidizing archaeon informs

physiological status of environmental populations. *ISME J.* **12**, 508–519 (2018).

11. Daebeler, A. et al. Cultivation and genomic analysis of "*Candidatus* Nitrosocaldus islandicus", an obligately thermophilic, ammonia-oxidizing Thaumarchaeon from a hot spring biofilm in Graendalur Valley, Iceland. *Front. Microbiol.* **9**, 193 (2018).
12. Abby, S. S. et al. *Candidatus* Nitrosocaldus cavascurensis, an ammonia oxidizing, extremely thermophilic archaeon with a highly mobile genome. *Front. Microbiol.* **9**, 28 (2018).
13. Daims, H. et al. Complete nitrification by *Nitrospira* bacteria. *Nature* **528**, 504–509 (2015).
