# Supplementary material for: Low yield and abiotic origin of N2O formed by the complete nitrifier Nitrospira inopinata
Source: Nat Commun. 2019 Apr 23;10:1836. doi: 10.1038/s41467-019-09790-x (PMC6478695; doi:10.1038/s41467-019-09790-x)
Supplement: Supplementary file 3 — Description of Additional Supplementary Files [file 41467_2019_9790_MOESM3_ESM.pdf]

Description of Additional Supplementary Files

File Name: Supplementary Data 1
Description: Source data for NOx metabolism gene inventory in publically available genomes of various nitrifiers. Each row corresponds to the genome record of one nitrifer. Columns A through G indicate the taxon, organism type, name, publication doi, and genome statistics (completeness, contamination, and strain heterogeneity) from each genome. Column H through AA indicate the locus tags (green filled cells) and copy number (blue filled cells) of each putative gene within each genome. Cells marked NA (not applicate) indicate the absence of a gene homolog from the corresponding genome.
