# Supplementary material for: Low yield and abiotic origin of N2O formed by the complete nitrifier Nitrospira inopinata
Source: Nat Commun. 2019 Apr 23;10:1836. doi: 10.1038/s41467-019-09790-x (PMC6478695; doi:10.1038/s41467-019-09790-x)
Supplement: Supplementary file 5 — Reporting Summary [file 41467_2019_9790_MOESM5_ESM.pdf]

nature research

Corresponding author(s): Michael Wagner

Last updated by author(s): Mar 17, 2019

Reporting Summary

Nature Research wishes to improve the reproducibility of the work that we publish. This form provides structure for consistency and transparency in reporting. For further information on Nature Research policies, see Authors & Referees and the Editorial Policy Checklist.

Statistics

For all statistical analyses, confirm that the following items are present in the figure legend, table legend, main text, or Methods section.

n/a	Confirmed	
☐	☒	The exact sample size (*n*) for each experimental group/condition, given as a discrete number and unit of measurement
☐	☒	A statement on whether measurements were taken from distinct samples or whether the same sample was measured repeatedly
☒	☐	The statistical test(s) used AND whether they are one- or two-sided<br>*Only common tests should be described solely by name; describe more complex techniques in the Methods section.*
☒	☐	A description of all covariates tested
☒	☐	A description of any assumptions or corrections, such as tests of normality and adjustment for multiple comparisons
☒	☐	A full description of the statistical parameters including central tendency (e.g. means) or other basic estimates (e.g. regression coefficient) AND variation (e.g. standard deviation) or associated estimates of uncertainty (e.g. confidence intervals)
☒	☐	For null hypothesis testing, the test statistic (e.g. *F*, *t*, *r*) with confidence intervals, effect sizes, degrees of freedom and *P* value noted<br>*Give P values as exact values whenever suitable.*
☒	☐	For Bayesian analysis, information on the choice of priors and Markov chain Monte Carlo settings
☒	☐	For hierarchical and complex designs, identification of the appropriate level for tests and full reporting of outcomes
☒	☐	Estimates of effect sizes (e.g. Cohen's *d*, Pearson's *r*), indicating how they were calculated

Our web collection on statistics for biologists contains articles on many of the points above.

Software and code

Policy information about availability of computer code

Data collection	SensorTrace Basic software (version 3.0) was used to log all of the micro-respirometry data. Prism software (version 8.0.1) was used to calculate the EC50 for data in Supplementary Figure 5.
Data analysis	Prism software (version 8.0.1) was used to calculate the EC50 in Supplementary Figure 5. Prism software was also used to calculate the standard deviations and means for all figures where standard deviations and center lines appear. MAFFT and FastTree2 software were used for the comparative genomic analysis (Figure 2).

For manuscripts utilizing custom algorithms or software that are central to the research but not yet described in published literature, software must be made available to editors/reviewers. We strongly encourage code deposition in a community repository (e.g. GitHub). See the Nature Research guidelines for submitting code & software for further information.

Data

Policy information about availability of data

All manuscripts must include a data availability statement. This statement should provide the following information, where applicable:

- Accession codes, unique identifiers, or web links for publicly available datasets
- A list of figures that have associated raw data
- A description of any restrictions on data availability

Genotype data in Figure 2 is uploaded as a part of the supplementary information. The manuscript does not contain any new protein, DNA, RNA, genetic polymorphism, macromolecular structure, microarray, or crystallographic data. The proteomics data in Supplementary Figure 3 is already publicly available under DOI https://doi.org/10.1038/nature16461

Field-specific reporting

Please select the one below that is the best fit for your research. If you are not sure, read the appropriate sections before making your selection.

☒ Life sciences ☐ Behavioural & social sciences ☐ Ecological, evolutionary & environmental sciences

For a reference copy of the document with all sections, see nature.com/documents/nr-reporting-summary-flat.pdf

Life sciences study design

All studies must disclose on these points even when the disclosure is negative.

Sample size	No statistical methods were used to predetermine sample size. All of the physiological experiments were performed three to six times (3 to 6 biological replicates).
Data exclusions	No data were excluded from the analyses.
Replication	All attempts at replication were successful and all biological replicates are displayed within the manuscript.
Randomization	Randomization was not applicable to this study. The emission of nitric oxide and nitrous oxide was measured in independent experiments from pure cultures of Nitrospira inopinata, Nitrospira moscoviensis, and Nitrosomonas europaea.
Blinding	Blinding was not applicable to this study.

Reporting for specific materials, systems and methods

We require information from authors about some types of materials, experimental systems and methods used in many studies. Here, indicate whether each material, system or method listed is relevant to your study. If you are not sure if a list item applies to your research, read the appropriate section before selecting a response.

Materials & experimental systems

n/a	Involved in the study
☒	☐ Antibodies
☒	☐ Eukaryotic cell lines
☒	☐ Palaeontology
☒	☐ Animals and other organisms
☒	☐ Human research participants
☒	☐ Clinical data

Methods

n/a	Involved in the study
☒	☐ ChIP-seq
☒	☐ Flow cytometry
☒	☐ MRI-based neuroimaging
